# Functional Analyses of Endometriosis-Related Polymorphisms in the Estrogen Synthesis and Metabolism-Related Genes

**DOI:** 10.1371/journal.pone.0047374

**Published:** 2012-11-06

**Authors:** Hsin-Shih Wang, Hsien-Ming Wu, Bi-Hwa Cheng, Chih-Feng Yen, Pi-Yueh Chang, Angel Chao, Yun-Shien Lee, Hsien-Da Huang, Tzu-Hao Wang

**Affiliations:** 1 Department of Obstetrics and Gynecology, Chang Gung Memorial Hospital, Lin-Kou Medical Center, Chang Gung University, Taoyuan, Taiwan; 2 Graduate Institute of Clinical Medical Sciences, College of Medicine, Chang Gung University, Taoyuan, Taiwan; 3 Department of Obstetrics and Gynecology, Chang Gung Memorial Hospital, Kaohsiung Medical Center, Kaohsiung, Taiwan; 4 Department of Laboratory Medicine, Chang Gung Memorial Hospital, Lin-Kou Medical Center, Chang Gung University, Taoyuan, Taiwan; 5 Genomic Medicine Research Core Laboratory, Chang Gung Memorial Hospital, Taoyuan, Taiwan; 6 Department of Biotechnology, Ming Chuan University, Taoyuan, Taiwan; 7 Department of Biological Science and Technology, Institute of Bioinformatics and Systems Biology, National Chiao Tung University, HsinChu, Taiwan; Clermont Université, France

## Abstract

Endometriosis is determined by genetic factors, and the prevalence of genetic polymorphisms varies greatly depending on the ethnic group studied. The objective of this study was to investigate the relationship between single nucleotide polymorphisms (SNPs) of 9 genes involved in estrogen biosynthesis and metabolism and the risks of endometriosis. Three hundred patients with endometriosis and 337 non-endometriotic controls were recruited. Thirty four non-synonymous SNPs, which change amino acid residues, were analyzed using matrix-assisted laser desorption-ionization time-of-flight mass spectrometry (MALDI-TOF MS). The functions of SNP-resulted amino acid changes were analyzed using multiple web-accessible databases and phosphorylation predicting algorithms. Among the 34 NCBI-listed SNPs, 22 did not exhibit polymorphism in this study of more than 600 Taiwanese Chinese women. However, homozygous and heterozygous mutants of 4 SNPs - rs6165 (genotype GG+GA, 307^Ala/Ala^+307^Ala/Thr^) of *FSHR*, rs 6166 (genotype GG+GA, 680^Ser/Asn^+680^Ser/Ser^) of *FSHR*, rs2066479 (genotype AA+AG, 289^Ser/Ser^+289^Ser/Gly^) of *HSD17B3* and rs700519 (genotype TT+TC, 264^Cys/Cys^+264^Cys/Arg^) of *CYP19*, alone or in combination, were significantly associated with decreased risks of endometriosis. Bioinformatics results identified 307^Thr^ of FSHR to be a site for O-linked glycosylation, 680^Ser^ of *FSHR* a phosphorylated site by protein kinase B, and 289^Ser^ of *HSD17B3* a phosphorylated site by protein kinase B or ribosomal protein S6 kinase 1. Results of this study suggest that non-synonymous polymorphisms of *FSHR*, *HSD17B3* and *CYP19* genes may modulate the risk of endometriosis in Taiwanese Chinese women. Identification of the endometrosis-preferential non-synonymous SNPs and the conformational changes in those proteins may pave the way for the development of more disease-specific drugs.

## Introduction

Endometriosis is a chronic, benign, estrogen-dependent disorder in women of reproductive age. It is characterized by the presence of ectopic endometrial tissue outside of the normal location (endometrial cavity) - mainly in the pelvic peritoneum, the ovaries, and the myometrium [1]. Clinical features of endometriosis include dysmenorrhea, deep dyspareunia, chronic pelvic pain, and infertility [2]. The development of endometriosis is regulated by enzymes and receptors that are involved in biosynthesis and metabolism of estrogens [1], [3], [4]. Therefore, inhibition of estradiol as the strategy of endometriosis therapy has been actively studied [5], [6]

**Figure 1:**
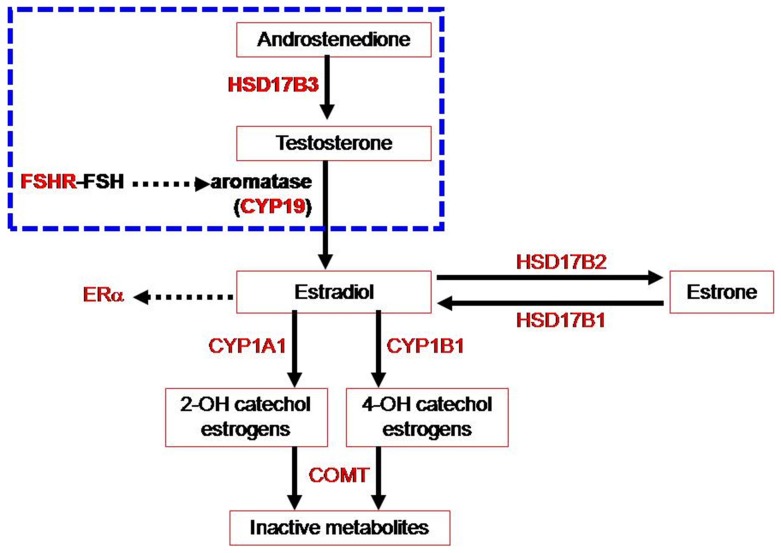
Nine genes that are involved in estrogen biosynthesis and metabolism. *Abbreviations:* COMT, catechol-O-methyl transferase; CYP1A1, cytochrome P450 1A1; CYP1B1, cytochrome P450 1B1; CYP19, cytochrome P450 19 ( = aromatase); ERα, estrogen receptor α; FSHR, follicle stimulating hormone receptor; HSD17B1, 17β-hydroxysteroid dehydrogenase I; HSD17B2, 17β-hydroxysteroid dehydrogenase II; HSD17B3, 17β-hydroxysteroid dehydrogenase III.

Estradiol, the most active form of estrogens, is produced either from testosterone catalyzed by aromatase (CYP19) or from estrone catalyzed by 17β-hydroxysteroid dehydrogenase type 1 (HSD17B1) (**Fig. 1**) [7]. In the human endometrium, inactivation of estradiol to estrone is induced by 17β-hydroxysteroid dehydrogenase type 2 (HSD17B2) [8]. The enzyme 17β-hydroxysteroid dehydrogenase type 3 (HSD17B3) converts androstenedione to testosterone, a precursor of estradiol [9]. In addition, two cytochrome P450 enzymes, cytochrome P450IAI (CYP1A1) and cytochrome P450IBI (CYP1B1), are responsible for the hydroxylation of 2-OH and 4-OH catechol estrogens which in turn induce DNA damage and mediate estrogen-induced carcinogenesis [10], [11]. Catechol-O-methyltransferase (COMT) inactivates 2-OH and 4-OH catechol estrogens by catalyzing the transfer of a methyl group from S-adenosyl-methionine to a hydroxyl group on a catechol nucleus [12].

The risk of endometriosis is related to genetic factors [13], [14]. Various single nucleotide polymorphisms (SNPs) have been associated with different susceptibilities to endometriosis [7], [15]–[18]. Our previous study has also shown that non-synonymous SNPs of FSH receptor gene [GG genotype (680^Ser/Ser^) and GA genotype (680^Ser/Asn^)] are related to a significantly lower risk of endometriosis [19]. HSD17B1 was also found to have profound species-related polymorphisms that resulted in different efficacies of steroid conversion during drug screening [20]. Collectively, endometriosis is thought to be determined by genetic background, and individual genetic variations that may interfere with local production and circulating levels of estrogen are likely to play roles in the development of endometriosis [21].

Matrix-assisted laser desorption-ionization (MALDI) was developed for ionizing and mass-analyzing large biomolecules [22]. In addition, matrix-assisted laser desorption-ionization time-of-flight mass spectrometry (MALDI-TOF MS) has been used for analysis of mini-sequencing products and SNP genotyping with advantages of time-saving, absolute results, and feasible automation for high throughput analysis [23]–[25].

Non-synonymous SNPs (nsSNPs) [26] may account for half of the known genetic variations linked to human inherited diseases [27]. Through changing amino acids of substrates or key flanking amino acids, nsSNPs may affect protein post-translational modifications (PTMs) such as phosphorylation and glycosylation. In the database dbPTM [28], [29], information of protein modifications and numerous amino acid variants associated with PTMs has been comprehensively compiled. dbPTM provides useful predictions about how non-synonymous SNPs may influence post-translational modifications of proteins. Additional computational methods, such as those used in KinasePhos [23]–[25], [30], can be used to study how non-synonymous SNPs influence protein phosphorylation by identifying kinase-specific protein phosphorylation sites in proteins.

In this study of more than 600 Chinese women, we have used MALDI-TOF MS to systemically genotype a total of 34 nsSNPs in genes that are involved in estrogen biosynthesis and metabolism. In addition to the characterization of 22 nsSNPs that exhibit a uniformed homozygosity unique to this Chinese population, we have identified the prevalence of genotypes of nsSNPs in FSHR at positions of 307 and 680. We also identified an association between mutant genotypes in FSHR, HSD17B3 and CYP19 and decreased risks of endometriosis. Results of bioinformatics analyses suggest the functional roles of such genetic variations in the related risk of endometriosis.

## Materials and Methods

### Subjects

Three hundred patients of ovarian endometrioma undergoing laparotomy or laparoscopy with further pathological confirmation at Chang Gung Memorial Hospital were included as previously described [19]. The scoring system revised by the American Society for Reproductive Medicine in 1997 was used to classify the stages of endometriosis. Another 337 postmenopausal women without any history of infertility, dysmenorrhea, endometriosis/adenomyosis, and surgeries for obstetrical/gynecological diseases were recruited to be healthy controls. All of the patients in the study were Taiwanese Chinese. Informed consents were obtained from all participants. The study was approved by the local Institutional Review Board **(**IRB#94–975B**)**. Blood samples (3 ml) were collected in heparinized tubes from all of patients in both groups [19]. Serum specimens were collected from another study, which was also approved by the local Institutional Review Board (IRB#98–1995A3).

### Non-synonymous Single Nucleotide Polymorphisms of Estrogen Synthesis and Metabolism-related Genes

Nine genes that regulate the biosynthesis and metabolism of estrogen (**Fig. 1**) were studied. They were *CYP19* (aromatase), *CYP1A1* (cytochrome P450 1A1), *CYP1B1* (cytochrome P450 1B1), *HSD17B1* (17β-hydroxysteroid dehydrogenase I), *HSD17B2* (17β-hydroxysteroid dehydrogenase II), *HSD17B3* (17β-hydroxysteroid dehydrogenase III), *ERα* (estrogen receptor α), *FSHR* (FSH receptor), and *COMT* (catechol-O-methyl transferase). A total of 34 nsSNPs, listed in **Table S1**, were chosen according to the database of National Center for Biotechnology Information (NCBI, www.ncbi.nlm.nih.gov/SNP/).

### Extraction of DNA

Genomic DNA from leukocyte in peripheral blood was extracted using a commercial kit, QIAmp DNA blood Midi Kit (Qiagen Inc., Valencia, CA, USA) according to the manufacturer’s recommendation.

### SNP Analysis by Matrix-assisted Laser Desorption-ionization Time-of-flight Mass Spectrometry (MALDI-TOF MS)

The MALDI-TOF MS SNP genotyping procedures were formatted for 96-wells [19], [31], [32]. Primers for the PCR and miniprimer extension reaction are shown in **Table S1**. Genomic regions spanning the respective SNP were amplified from each sample DNA. PCR amplification was performed in a final volume of 10 μl containing 5 ng of genomic DNA, 1X PCR buffer, 100 μM each of dTTP, dATP, dCTP, and dGTP, 1 μM each of primers, and 1U Taq DNA polymerase followed by 3 min denaturation at 95°C and 40 cycles of denaturation at 95°C for 30 sec, annealing at Tm of each primer set for 30 sec, extension at 72°C for 30 sec, and final extension at 72°C for 2 min. All thermal cycles were run on a thermocycler (MJ Research, Watertown, MA, USA). Amplified double stranded DNA was isolated using GenoPure DS purification kit (Bruker Daltonics, Bremen, Germany) with automated liquid handlers, MAP-II8 and PureDisk (Bruker Daltonics, Bremen, Germany).

Allele-specific primer extension reaction was catalyzed by ThermoSeqencase (Amersham Pharmacia, Amersham, UK) at 94°C for 8 sec, 52°C for 8 sec, and 72°C for 8 sec, for 50 cycles. Primer extension products were treated with GenoPure Oligo purification kit (Bruker Daltonics, Bremen, Germany) to remove salts in the reaction buffer.

The matrix 3-hydroxypicolinic acid (3-HPA) (Fluka, Buchs, Switzerland) was used in a concentration of 10 mg/ml containing 1 mg/ml di-ammonium hydrogene citrate. Half μl of matrix was first spotted with the MAP II/8 robotic system on the AnchorChip and allowed to dry, and then 0.5 μl of primer extension product was loaded to the dried matrix. Finally, 0.5 μl of 75% acetonitrile was added to the sample followed by MALDI-TOF MS (Autoflex, Bruker Daltonics, Germany) analysis.

### Measurement of Serum Estradiol (E2)

Serum estradiol levels were assayed with the electrochemiluminescence immunoassay "ECLIA" on Elecsys and cobas e Immunoassay analyzer (Roche, Basel, Switzerland) in a College of American Pathologist (CAP)-certified laboratory. The estradiol assay sensitivity was 5 pg/ml, and the intra- and inter-assay coefficients of variation were 1.8% and 6.2%, respectively.

### Statistical Analysis

The Chi-square (χ^2^) test was used to compare genotype distributions between patients with endometriosis and controls. Hardy-Weinberg equilibrium was examined using a goodness-of-fit χ^2^ test with one degree of freedom in order to compare the observed genotype frequencies with the expected genotype frequencies among study subjects. The dominant effect was analyzed by comparing one homozygous genotype (MM) to the summation of the other two genotypes - the heterozygous (MN) and the other homozygous (NN). The statistical modeling of univariate logistic regression was used to calculate the odds ratio (OR) of genetic effects. Statistical analyses were conducted by the Statistical Analysis System (SAS) software (version 8.1 for windows; SAS Institute Inc., Cary, NC). A *P* value of<0.05 was considered statistically significant. Results are presented as OR and the 95% confidence interval (CI).

### Functional Network Analysis of Key Proteins

Procedures of networks analysis were similar to what we previously reported [33]–[35]. Briefly, we used the “analyze networks” algorithm in MetaCore (GeneGo, St. Joseph, MI) to build the networks that consisted of FSHR, HSD17B3 and CYP19. MetaCore includes a curated database of human protein interactions and metabolism; thus, it is useful for analyzing a cluster of genes in the context of regulatory networks and signaling pathways [36]. For the network analysis of a group of genes, MetaCore can be used to calculate the statistical significance (*p* value) based on the probability of assembly from a random set of nodes (genes) of the same size as the input list [36].

### Computational Analysis of Non-synonymous SNPs for their Effects on Post-translational Modification

To examine how nsSNPs affect post-translational protein modifications leading to changes in estrogen synthesis and metabolism, we looked into multiple databases that were previously reported [28]. Protein annotations were obtained from UniProt [37], which is a repository of protein properties. Information on protein glycosylation and phosphorylation associated with non-synonymous SNPs were obtained from dbPTM [29]. To identify the protein phosphorylation sites associated with non-synonymous SNPs, we adopted a well tested method - KinasePhos [23]–[25], [30] to identify kinase-specific phosphorylation sites against amino acids changed by non-synonymous SNPs. KinasePhos, which is a computational tool developed by our group based on Hidden Markov models, can accurately identify kinase-specific protein phosphorylation sites [23]. Taking the polymorphism of FSH receptor gene (*FSHR*) (Asn680Ser caused by A→G) as an example, 680^Ser^ but not 680^Asn^ in FSHR might be a potential phosphorylation site. Thus, the phosphorylation status of amino acids 680 may be affected by the polymorphism. In this study, all of the significant disease-associated non-synonymous SNPs were computationally analyzed for their influences on protein phosphorylation and glycosylation.

## Results

### Demographics of Studied Groups

Ages of patients with endometriosis ranged from 21 to 42 years (mean age, 34.3 years) whereas the ages of normal controls ranged from 45 to 61 years (mean age, 52.2 years). Body mass indices (kg/m^2^) of both groups were similar (ranged from 16.7 to 30.9 for patients with endometriosis and from 17.2 to 31.6 for normal controls) (**Table 1**). Stages of endometriosis were classified according to the revised scoring system proposed by American Society for Reproductive Medicine. Most patients with endometriosis recruited in the present study were in advanced stages (III and IV, 80.4%) (**Table 1**). Women with endometriosis had a significant (*P*<0.00001) lower parity than healthy controls. Additionally, endometriosis patients who were older than 37 years when they first sought medical treatment had a significantly higher parity than those who first sought medical treatment younger than 37 years (*P*<0.00001) (**Table 1**).

**Table 1:** Characterization of the studied population.

	Cases of endometriosis	Normal controls	*P* *
	(n = 300)	(n = 337)	
Age	34.3±6.9	52.2±4.2	<0.00001
Body mass index (kg/m^2^)	22.0±3.4	23.6±3.1	NS
Parity	1.1±1.1	2.6±1.1	<0.00001
>37 years in endometriosis (n = 124)	1.6±1.1^#^		<0.00001^#^
<37 years in endometriosis (n = 176)	0.7±1.0^#^		
Stage of endometriosis			
I	2.3% (n = 7)		
II	17.3% (n = 52)		
III	55.0% (n = 165)		
IV	25.4% (n = 76)		

* Student-t test.

NS: not significant.

Age differences between endometriosis group (34.3±6.9) and controls (52.2±4.2) might partly account for the parity difference. In addition, this parity difference might reflect a higher incidence of infertility in endometriosis patients. Moreover, our data also suggested that endometriosis-related infertility was correlated with the age at diagnosis, as older endometriosis group (>37 years old) who sought medical treatment for the first time had a significantly higher parity than younger ones (*P*<0.00001) (**Table 1**). These findings implied that patients with earlier onset of endometriosis suffered more from infertility.

### Serum Estradiol (E2) Levels in Patients with Surgically Confirmed Endometriosis Before and After Operation

In an independent retrospective study, serum estradial levels in a cohort of 100 patients before and after operation were measured. Sera obtained from 100 age-matched women were measured as controls. There was not significant difference in serum levels of estradiol in these three groups (**Table S1**).

### Chinese Preferential Homozygosity of Non-synonymous SNPs in Estrogen Synthesis and Metabolism-related Genes

Among 34 nsSNPs genotyped in this study (**Table S2**), 22 were found to be homozygous in more than 600 Taiwanese Chinese women. These 22 SNPs were *CYP19* (rs2304462, GG homozygous; rs1803154, AA homozygous), *CYP1A1* (rs1799814, CC homozygous; rs2229150, CC homozygous; rs2278970, GG homozygous; rs2856833, CC homozygous; rs4987133, GG homozygous), *CYP1B1* (rs1800440, AA homozygous; rs4398252, TT homozygous; rs4986887, GG homozygous; rs4986888, CC homozygous), *HSD17B2* (rs8191136, GG homozygous), *HSD17B3* (rs2066480, GG homozygous), *ERα* (rs9340773, GG homozygous; rs17847065, CC homozygous; rs17847076, AA homozygous), *FSHR* (rs6167, CC homozygous; rs1126714, CC homozygous), and *COMT* (rs6270, GG homozygous; rs6267, GG homozygous; rs5031015, GG homozygous; rs4986871, CC homozygous).

### Mutant SNPs of HSD17B3 and CYP19 Showed a Lower Risk in Endometriosis Patients Younger than 37 Years

In all endometriosis patients (n = 300), a univariate analysis for the Gly289Ser (G/A) polymorphism of 17β-hydroxysteroid dehydrogenase III (*HSD17B3*) revealed that only heterozygous mutant SNP (genotype AG, 289^Ser/Gly^) of *HSD17B3* showed a significantly decreased risk of endometriosis (*P* = 0.03; OR = 0.7) as compared to the controls (n = 337) (**Table 2**). In contrast, in endometriosis patients younger than 37 years (n = 176), combined homozygous and heterozygous mutant SNP (genotype AA+AG, 289^Ser/Ser^+289^Ser/Gly^) of *HSD17B3* showed a significantly lower risk of endometriosis (*P* = 0.007; OR = 0.59) in comparison with the controls (n = 337) (**Table 3**). In this setting, both heterozygous mutant SNP (genotype AG, 289^Ser/Gly^) and homozygous mutant SNP (genotype AA, 289^Ser/Ser^) of *HSD17B3* showed a significantly decreased risk of endometriosis (*P* = 0.005; OR = 0.57) and *P* = 0.02; OR = 0.69), respectively, as compared to the controls (n = 337).

**Table 2:** Genotype frequency and overall association with estrogen synthesis and metabolism-related genes for women with endometriosis (n = 300) and controls (n = 337).

Genes	SNP ID. No.			Genotypes	Cases	(%)	Controls	(%)	OR	95% CI	*P* *
*CYP1A1*	rs1048943	A: Ile	Ile462Val (A→G)	AA	167	(55.7)	182	(54.0)	1.00	Ref.	-
		G: Val		AG	110	(36.7)	134	(39.8)	0.90	0.64–1.26	0.51
				GG	23	(7.7)	21	(6.2)	0.99	0.77–1.29	0.97
				AG+GG	133	(44.3)	155	(46.0)	0.94	0.67–1.29	0.67
*CYP1A1*	rs4646422	G: Gly	Gly45Asp (G→A)	GG	214	(71.3)	251	(74.5)	1.00	Ref.	–
		A: Asp		AG	81	(27.0)	79	(23.4)	1.18	0.69–2.13	0.50
				AA	5	(1.7)	7	(2.1)	1.09	0.69–1.82	0.68
				AG+AA	86	(28.7)	86	(25.5)	1.15	0.68–2.03	0.56
*CYP1B1*	rs10012	C: Arg	Arg48Gly (C→G)	CC	202	(67.3)	238	(70.6)	1.00	Ref.	–
		G: Gly		CG	86	(28.7)	92	(27.3)	1.09	0.65–1.89	0.73
				GG	12	(4.0)	7	(2.1)	1.20	0.78–1.92	0.39
				CG+GG	98	(32.7)	99	(29.4)	1.16	0.70–1.97	0.54
*CYP1B1*	rs1056827	G: Ala	Ala119Ser (G→T)	GG	202	(67.3)	238	(70.6)	1.00	Ref.	-
		T: Ser		GT	86	(28.7)	92	(27.3)	1.09	0.65–1.89	0.73
				TT	12	(4.0)	7	(2.1)	1.20	0.78–1.92	0.39
				GT+TT	98	(32.7)	99	(29.4)	1.16	0.70–1.97	0.54
*CYP1B1*	rs1056836	C: Leu	Leu432Val (C→G)	CC	249	(83.0)	271	(80.0)	1.00	Ref.	–
		G: Val		CG	51	(17.0)	63	(19.1)	0.88	0.57–1.35	0.54
				GG	0	(4.0)	3	(0.9)	0.82	0.55–1.21	0.29
				CG+GG	51	(17.0)	66	(20.0)	0.84	0.55–1.28	0.40
*CYP19*	rs700519	C: Arg	Arg264Cys (C→T)	CC	222	(74.0)	242	(71.8)	1.00	Ref.	–
		T: Cys		CT	36	(12.0)	28	(8.3)	1.40	0.80–2.47	0.21
				TT	42	(14.0)	67	(19.9)	0.79	0.60–1.04	0.08
				CT+TT	78	(26.0)	95	(28.2)	0.90	0.62–1.29	0.54
*CYP19*	rs2236722	T: Trp	Trp39Arg (T→C)	TT	265	(88.3)	304	(90.0)	1.00	Ref.	–
		C: Arg		TC	34	(11.3)	31	(9.0)	1.22	0.56–3.12	0.55
				CC	1	(0.3)	2	(0.6)	0.79	0.61–3.19	0.46
				TC+CC	35	(11.6)	33	(9.6)	0.90	0.58–3.21	0.50
*HSD17B1*	rs605059	G: Gly	Gly313Ser (G→A)	GG	81	(27.0)	94	(27.9)	1.00	Ref.	-
		A: Ser		AG	166	(55.3)	175	(51.9)	1.10	0.75–1.61	0.61
				AA	53	(17.7)	68	(20.2)	0.97	0.77–1.22	0.77
				AG+AA	219	(73.0)	243	(72.1)	1.05	0.73–1.51	0.80
*HSD17B2*	rs8191246	A: Thr	Thr388Trp (A→G)	AA	280	(93.3)	304	(90.2)	1.00	Ref.	–
		G: Trp		AG	20	(0.07)	32	(9.5)	0.68	0.36–1.26	0.20
				GG	0	(0)	1	(0.3)	0.66	0.35–1.18	0.16
				AG+GG	20	(0.07)	33	(9.8)	0.66	0.35–1.21	0.19
*HSD17B3*	rs2066479	G: Gly	Gly289Ser (G→A)	GG	186	(62.0)	186	(55.2)	1.00	Ref.	–
		A: Ser		AG	94	(31.3)	135	(40.1)	0.70	0.49–0.98	0.03
				AA	20	(6.7)	16	(4.8)	0.87	0.67–1.14	0.31
				AG+AA	114	(38.0)	151	(44.8)	0.76	0.54–1.05	0.08
*FSHR*	rs6165	A: Thr	Thr307Ala (A→G)	AA	140	(46.7)	156	(46.3)	1.00	Ref.	-
		G: Ala		AG	122	(40.7)	135	(40.0)	0.73	0.52–1.04	0.07
				GG	38	(12.7)	46	(13.7)	0.85	0.67–1.08	0.17
				AG+GG	160	(53.3)	181	(53.7)	0.75	0.54–1.05	0.08
*FSHR*	rs6166#	A: Asn	Asn680Ser (A→G)	AA	148	(49.3)	126	(37.4)	1.00	Ref.	–
		G: Ser		AG	121	(40.3)	173	(51.0)	0.60	0.42–0.84	0.002
				GG	31	(10.3)	38	(11.3)	0.75	0.59–0.95	0.02
				AG+GG	152	(50.7)	211	(62.6)	0.61	0.44–0.86	0.002
*COMT*	rs4680	G: Val	Val158Met (G→A)	GG	171	(57.0)	194	(57.6)	1.00	Ref.	–
		A: Met		AG	111	(37.0)	116	(34.4)	1.09	0.77–1.53	0.63
				AA	18	(6.0)	27	(8.0)	0.96	0.74–1.25	0.77
				AG+AA	129	(43.0)	143	(42.4)	1.02	0.74–1.42	0.89

SNP, single nucleotide polymorphism; OR, odds ratio; CI, confidence interval.

*CYP1A1*, cytochrome P450IAI; *CYP1B1*, cytochrome P450IBI; *CYP19*, aromatase; *HSD17B1*, 17β-hydroxysteroid dehydrogenase I; *HSD17B2*, 17β-hydroxysteroid dehydrogenase II; *HSD17B3*, 17β-hydroxysteroid dehydrogenase III; *FSHR*, FSH receptor;

*COMT*, catechol-O-methyl transferase.

* Chi-square test.

# Data used were published previously in Wang *et al.*, 2011 [19].

**Table 3:** Genotype frequency of single nucleotide polymorphisms and overall association with estrogen synthesis and metabolism-related genes for women with endometriosis aged younger than 37 years (n = 176) and controls (n = 337).

Genes	SNP ID. No.			Genotypes	Cases	(%)	Controls	(%)	OR	95% CI	*P* *
*HSD17B3*	rs2066479	G: Gly	Gly289Ser (G→A)	GG	119	(68.6)	186	(55.2)	1.00	Ref.	–
		A: Ser		AG	49	(27.8)	135	(40.1)	0.57	0.39–0.86	0.005
				AA	8	(4.6)	16	(4.8)	0.69	0.49–0.96	0.02
				AG+AA	57	(32.4)	151	(44.8)	0.59	0.39–0.88	0.007
*CYP19*	rs700519	C: Arg	Arg264Cys (C→T)	CC	132	(75.0)	242	(71.8)	1.00	Ref.	–
		T: Cys		CT	24	(13.6)	28	(8.3)	1.57	0.83–2.94	0.13
				TT	20	(11.4)	67	(19.9)	0.71	0.50–0.98	0.03
				CT+TT	44	(25.0)	95	(28.2)	0.85	0.55–1.31	0.44

SNP, single nucleotide polymorphism; OR, odds ratio; CI, confidence interval.

*HSD17B3*, 17β-hydroxysteroid dehydrogenase III; *CYP19*, aromatase.

* Chi-square test.

Similarly, in younger patients with endometriosis (n = 176), homozygous mutant SNP of *CYP19* (genotype TT, 264^Cys/Cys^) showed a significantly lower risk of endometriosis (*P* = 0.03; OR = 0.71) when compared to the controls (n = 337) (**Table 3**). This result was not shown when both younger and older groups were combined (n = 300) (**Table 2**).

### A SNP of FSHR at Position 680 in Combination with SNPs in HSD17B3, CYP19 or FSHR at Position 307 showed Decreased Risks of Endometriosis

In regard to the Asn680Ser (A/G) polymorphism of *FSHR* and the Gly289Ser (G/A) polymorphism of *HSD17B3*, univariate analyses on all endometriosis women (n = 300) revealed that a combination of homozygous/heterozygous mutants of *FSHR* (genotype GG+GA, 680^Ser/Ser^+680^Ser/Asn^) and homozygous/heterozygous mutants of *HSD17B3* (genotype AA+AG, 289^Ser/Ser^+289^Ser/Gly^) was associated with significantly decreased risk of endometriosis (*P* = 0.00002; OR = 0.46) as compared to the combination of homozygous wild types of *FSHR* and *HSD17B3* **(** **Table 4** **)**. Similarly, a significantly decreased risk for endometriosis was found in women who had at least a mutant allele of *FSHR* (genotype GG+GA, 680^Ser/Ser^+680^Ser/Asn^) and *CYP19* (genotype TT+TC, 264^Cys/Cys^+264^Cys/Arg^) (*P* = 0.01; OR = 0.66) **(** **Table 4** **)**. Furthermore, a significantly decreased risk for endometriosis was also observed in women who had at least a mutant allele in *FSHR* at position 680 (genotype GG+GA, 680^Ser/Ser^+680^Ser/Asn^) and position 307 (genotype GG+GA, 307^Ala/Ala^+307^Ala/Thr^) (*P* = 0.01; OR = 0.66) **(** **Table 4** **)**.

**Table 4:** Combined genotypes of two single nucleotide polymorphisms (mutant, homozygous) in women with endometriosis (n = 300) and controls (n = 337).

Combined genotypes of two SNPs	Cases	(%)	Controls	(%)	OR**	95% CI**	*P* *
*FSHR* # (rs6166, AA)+*HSD17B3* (rs2066479, GG)	99	(33.0)	62	(18.4)	1.00	Ref.	
*FSHR* # (rs6166, GG+GA)+*HSD17B3* (rs2066479, AA+AG)	201	(67.0)	275	(81.6)	0.46	0.31–0.67	0.00002
*FSHR* # (rs6166, AA)+*CYP19* (rs700519, CC)	109	(36.3)	92	(27.3)	1.00	Ref.	
*FSHR* # (rs6166, GG+GA)+*CYP19* (rs700519, TT+TC)	191	(63.7)	245	(72.7)	0.66	0.46–0.93	0.01
*FSHR* # (rs6166, AA)+*FSHR* (rs6165, AA)	134	(44.7)	117	(34.7)	1.00	Ref.	
*FSHR* # (rs6166, GG+GA)+*FSHR* (rs6165, GG+GA)	166	(55.3)	220	(65.3)	0.66	0.47–0.92	0.01
*FSHR* # (rs6166, AA)+*COMT* (rs4680, GG)	90	(30.0)	72	(21.4)	1.00	Ref.	
*FSHR* # (rs6166, GG+GA)+*COMT* (rs4680, AA+AG)	210	(70.0)	265	(78.6)	0.63	0.43–0.92	0.01

SNP, single nucleotide polymorphism; OR, odds ratio; CI, confidence interval.

*FSHR* (rs6166, AA), 680^Asn/Asn^; *FSHR* (rs6166, GG+GA), 680^Ser/Ser^+680^Ser/Asn^.

*HSD17B3* (rs2066479, GG), 289^Gly/Gly^; *HSD17B3* (rs2066479, AA+AG), 289^Ser/Ser^+289^Ser/Gly^.

*CYP19* (rs700519, CC), 264^Arg/Arg^; *CYP19* (rs700519, TT+TC), 264^Cys/Cys^+264^Cys/Arg^.

*FSHR* (rs6165, AA), 307^Thr/Thr^; *FSHR* (rs6165, GG+GA), 307^Ala/Ala^+307^Ala/Thr^.

*COMT* (rs4680, GG), 158^Val/Val^; *COMT* (rs4680, AA+AG), 158^Met/Met^+158^Met/Val^.

* Chi-square test.

** Calculation was performed following a dominant genotype model (MM of combined SNPs compared with [NN+NM] of combined SNPs).

# Data used were published previously in Wang *et al.*, 2011 [19].

### Two Non-synonymous SNPs of FSHR at Positions 307 (rs6165) and 680 (rs6166) in Chinese Women were not in the Same Haplotype

In all women studied, frequencies of genotype with combined homozygous SNPs in FSHR at positions 307 (rs6165) and 680 (rs6166) (both wild-type alleles, 307^Thr/Thr^680^Asn/Asn^, and both mutant alleles, 307^Ala/Ala^680^Ser/Ser^) were 39.4% and 9.6% respectively. In contrast, 38.8% of women studied possessed genotypes of combined heterozygous SNPs in FSHR (307^Ala/Thr^680^Ser/Asn^) **(** **Table 5** **)**. Furthermore, at least a heterozygous SNP in FSHR at positions 307 (rs6165) and 680 (rs6166) was found in 51.0% of Taiwanese Chinese women studied. These results indicated that, even though these 2 SNPs reside in the exons of the same gene, they are not in the same haplotype.

**Table 5:** Non-synonymous SNPs of FSHR at positions 307 (rs6165) and 680 (rs6166) in Chinese women.

FSHR (rs6165)	FSHR (rs6166)	Cases of endometriosis (n = 300)	Controls (n = 337)	Cases+Controls (n = 637)	%
307^Thr/Thr^	680^Asn/Asn^	134	117	251	39.4
307^Ala/Thr^	680^Ser/Asn^	111	136	247	38.8
307^Ala/Ala^	680^Ser/Ser^	28	33	61	9.6
307^Thr/Thr^	680^Ser/Asn^	5	33	38	6.0
307^Ala/Ala^	680^Asn/Asn^	5	10	15	2.3
307^Ala/Thr^	680^Asn/Asn^	9	3	12	1.9
307^Ala/Ala^	680^Ser/Asn^	5	3	8	1.3
307^Ala/Thr^	680^Ser/Ser^	2	1	3	0.5
307^Thr/Thr^	680^Ser/Ser^	1	1	2	0.3

*FSHR* (rs6165), AA: 307^Thr/Thr^; GG: 307^Ala/Ala^; GA: 307^Ala/Thr^.

*FSHR* (rs6166), AA: 680^Asn/Asn^; GG: 680^Ser/Ser^; GA: 680^Ser/Asn^.

### Functional Networks Among FHSR, HSD17B3 and CYP19

**Figure 2:**
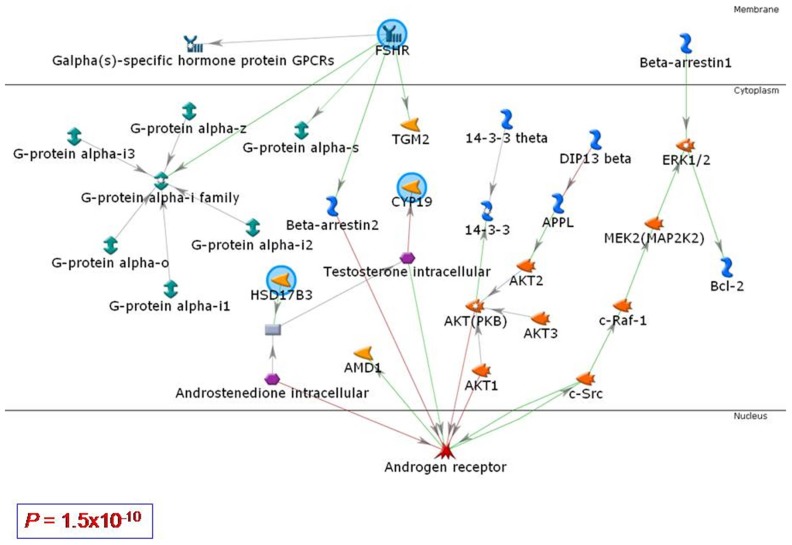
Network analysis for the functional interactions among FSHR, CYP19 and HSD17B3. The networks of signaling pathways were built using MetaCore (GeneGo Inc.,). Green lines indicate stimulation, and red lines indicate inhibition. *Abbreviations:* 14-3-3 theta, tyrosine 3-monooxygenase/tryptophan 5-monooxygenase activation protein, theta polypeptide; AKT, v-akt murine thymoma viral oncogene homolog 1; APPL, adaptor protein, phosphotyrosine interaction, PH domain and leucine zipper containing 1; DIP13 beta, adaptor protein, phosphotyrosine interaction, PH domain and leucine zipper containing 2; ERK2, extracellular signal-regulated kinase 2; FSHR, follicle stimulating hormone receptor; HSD17B3, 17β-hydroxysteroid dehydrogenase III; CYP19, cytochrome P450 19 ( = aromatase); MTA2, metastasis associated 1 family, member 2; c-Src, v-src sarcoma (Schmidt-Ruppin A-2) viral oncogene homolog (avian); TGM2, transglutaminase 2.

Using MetaCore algorithm for networks analysis, we found that FSHR, HSD17B3 and CYP19 interacted in the network of pathways with a *P* value of 1.5×10^−10^ **(** **Fig. 2** **)**, indicating that the probability of assembly from random sets of nodes (genes) was very low [38]. The pathways of FHSR and those of HSD17B3 and CYP19 intersected at the androgen receptor (AR).

### Modification of Protein Glycosylation and Phosphorylation by Non-synonymous SNPs

**Figure 3:**
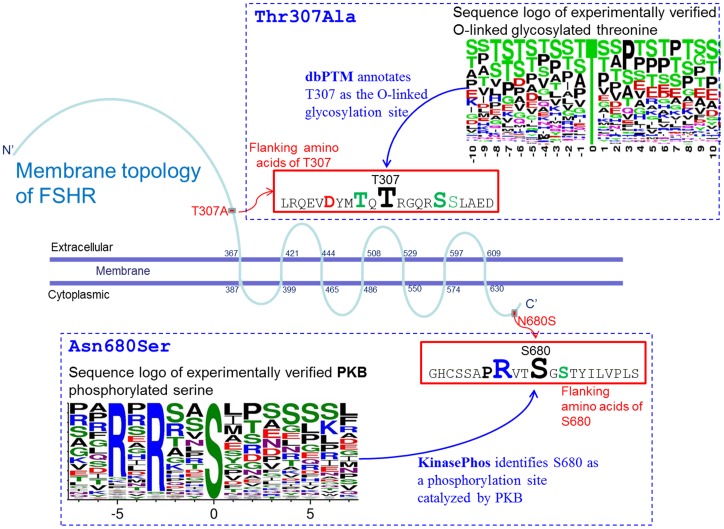
The O-linked glycosylated 307^Thr^ and phosphorylated 680^Ser^ in FSHR. dbPTM is a database for post-translational modification (http://dbptm.mbc.nctu.edu.tw/), and KinasePhos is a web-based tool to computationally predict phosphorylation sites within given protein sequences (http://kinasephos.mbc.nctu.edu.tw/).

**Figure 3** depicts the membrane topology of FSHR, an O-linked glycosylated amino acids at 307^Thr^, and a phosphorylated amino acids at 680^Ser^. Based on the statistics of UniProt membrane proteins, 212 of 216 O-linked glycosylation sites occur at extracellular regions in 49 membrane proteins [37]. Thus, FSHR 307^Thr^ (wild type), which is located extracellularly, was identified as an O-linked glycosylation site by dbPTM (See http://dbptm.mbc.nctu.edu.tw/search_result.php?search_type=db_id&swiss_id=FSHR_HUMAN, and http://www.uniprot.org/uniprot/P23945). The flanking sequence of 307^Thr^ has a similar composition of amino acids to those experimentally verified O-linked glycosylated threonine. A sequence logo [39] is presented to illustrate the amino acids composition of O-linked glycosylation substrate (**Fig. 3**).

Using KinasePhos [23], [25], FSHR 680^Ser^ (mutant) was identified as a phosphorylation site that may be catalyzed by protein kinase B (PKB), protein kinase A (PKA), or ribosomal protein S6 kinase (RSK) (See http://ca.expasy.org/cgi-bin/variant_pages/get-sprot-variant.pl?VAR_013905 and http://ca.expasy.org/cgi-bin/variant_pages/get-sprot-variant.pl?VAR_013903). According to KinasePhos, an arginine (R) at the -3 position of 680^Ser^ of FSHR (**Fig. 3**) is similar to the motif of PKB phosphorylated serine, which requires an arginine (R) at -3 position. Likewise, HSD17B3 289^Ser^ (mutant) was also identified to be a phosphorylation site that can be catalyzed by PKB or RSK1 (See http://dbptm.mbc.nctu.edu.tw/search_result.php?search_type=db_id&swiss_id=DHB3_HUMAN, http://www.uniprot.org/uniprot/P37058, and http://ca.expasy.org/cgi-bin/variant_pages/get-sprot-variant.pl?VAR_014871).

## Discussion

It is important to choose appropriate controls in association studies, because selection bias adversely affects results [40]. Previously, control groups have been selected from female newborns from the same ethnic group as the population control [41] or the women drawn from the same clinic population who were free of endometriosis [40]. Women with laparoscopic confirmation of free of endometriosis seem to be rational controls; however, such groups may develop endometriosis later in the life. Although laparoscopy remains the gold standard approach to confirm endometriosis [42], this invasive procedure is never done without medical indications, such as chronic pelvic pain and adnexal masses. Such potential co-morbilities may exclude these women from healthy controls. Therefore, the non-endometriosis controls in this study were chosen from postmenopausal women aged 45 years or older (range: 45–61 years; mean: 52.2 years), who had no history of infertility and dysmenorrhea, no previous diagnosis of endometriosis and/or adenomyosis, and no surgical history for obstetrical or gynecological diseases. Accordingly, we did not adjust the odds ratios by age between endometriosis cases and controls in this study.

Several SNPs of the estrogen synthesis- and metabolism-related genes, such as *CYP19* [7], [15], [18], *CYP1A1* and *COMT* [43], and *CYP1B1* [44], have been found to be associated with increased risk of endometriosis. However, among the 35 NCBI-listed non-synonymous SNPs (including a SNP [rs6166] that was published previously in [19]), twenty two in eight genes (*CYP19*, *CYP1A1*, *CYP1B1*, *HSD17B2*, *HSD17B3*, *ERα*, *FSHR*, *COMT)* were found to be homozygous in our study of Taiwanese women **(Table S2)**. These findings indicated that ethnic factors were important for different SNP prevalence among different geographic populations.

Commonly observed in clinics, the occurrence of endometriosis in young patients is frequently complicated by a higher rate of infertility. We found that endometriosis patients who were younger than 37 years when they first sought medical treatment had a significantly lower parity (**Table 1**). The selection of 37 years of age was based on the identification of accelerated disappearance of human ovarian follicles at the age of 37 years [45], [46]. The mutant SNP at position 289 of *HSD17B3* in at least one allele may protect Taiwanese Chinese women (especially younger than 37 years) from early-onset, severe endometriosis and further preserve the fertility.

Serum levels of estradiol (E2) in patients with endometriosis are rarely compared between the periods before and after surgical removal of endometriotic tissues. We did not find that operation caused significant change of serum estradiol. There is not difference in serum estradiol levels between patients with endometriosis and controls either (**Table S1**). The development of endometriosis is currently correlated with overproduction of local estrogen by increased aromatase activity in the endometriotic tissue [1], [47]. Therefore, it is advisable to localize the following molecular mechanisms of estrogen production and metabolism only to endometriotic tissues.

A combination of homozygous/heterozygous mutants of *HSD17B3* (genotype AA+AG, 289^Ser/Ser^+289^Ser/Gly^) and homozygous/heterozygous mutants of *FSHR* (genotype GG+GA, 680^Ser/Ser^+680^Ser/Asn^) was associated with a significantly decreased risk of endometriosis (*P* = 0.00002) in comparison with the combination of homozygous wild-type SNPs of *HSD17B3* and *FSHR* **(** **Table 4** **)**. mRNA expression levels of HSD17B3 were shown to be higher in subjects with GG polymorphism (wild-type, 289^ Gly/Gly^) than those with SS polymorphism (mutant, 289^Ser/Ser^), indicating that homozygous HSD17B3 with GG polymorphism (289^ Gly/Gly^) has a higher enzyme activity [48]. In addition, the wild-type homozygous polymorphism of *FSHR* gene (680^Asn/Asn^) induces higher aromatase activity than mutant *FSHR* gene, resulting in production of more estrogens and stimulating proliferation of endometriotic tissues [19]. Collectively, the GG homozygous genotype of polymorphism of *HSD17B3* (289^Gly/Gly^) (wild type) may play a crucial role in the development of endometriosis in the presence of AA homozygous genotype of polymorphism of *FSHR* (680^Asn/Asn^) (wild type).

In the present study, the frequencies of completely combined homozygous SNPs in FSHR at positions 307 and 680 (307^Thr/Thr^680^Asn/Asn^, wild-type homozygous, and 307^Ala/Ala^680^Ser/Ser^, mutant homozygous) were 39.4% and 9.6% while the frequency of at least a heterozygous SNP in FSHR at positions 307 and 680 was 51.0% **(** **Table 5** **)**. These findings were similar to those in a Japanese population reported previously [49], [50]. Clinically, the 680^Ser^ allele was associated with lower sensitivity to FSH during ovulation induction [51]. Similarly, a higher dose of exogenous FSH is required to achieve ovulation induction in women with FSHR genotype 307^Ala/Ala^680^Ser/Ser^ (mutant homozygous) [49], [50]. In addition, FSHR with alleles 307^Thr/Thr^680^Asn/Asn^ (wild-type homozygous) possesses higher bioactivity of intracellular transduction and aromatase after binding to FSH, since women with genotype 307^Thr/Thr^680^Asn/Asn^ are more likely to develop severe ovarian hyperstimulation syndrome (OHSS) during ovulation induction with FSH [52]. In summary, FSHR with completely wild-type homozygous SNPs at positions 307 and 680 (307^Thr/Thr^680^Asn/Asn^) had higher sensitivity to FSH and an increased risk of endometriosis, whereas FSHR possessing at least an allele of mutant SNP at positions 307 and 680 lower risk of endometriosis.

Non-synonymous SNP in *CYP19* gene (Arg264Cys, C→T) alone was not correlated with the change in risk of endometriosis (**Table 2**). However, in the presence of mutant SNP in *FSHR* gene (680^Ser/Ser^+680^Ser/Asn^), mutant SNPs in *CYP19* gene demonstrated a significantly decreased risk of endometriosis (**Table 4**). The binding of FSH to FSHR activates aromatase (CYP19), which in turn induces estrogen production [7]. In the presence of mutant SNP in *FSHR* gene (680^Ser/Ser^+680^Ser/Asn^), mutant SNPs in *CYP19* gene demonstrated a significantly decreased risk of endometriosis (**Table 4**), indicating that FSHR and CYP19 had synergistic effects on the production of estrogen. On the contrary, the COMT polymorphism (rs4680) was not associated with the risk of endometriosis, which is in agreement with a recent report of a Brazilian population [53].

Revealed by network analysis using MetaCore (**Fig. 2**), the interaction among FSHR, CYP19 and HSD17B3 at the androgen receptor (AR) may have a clinical importance. Active androgens and AR are shown in endometriotic lesion of women with stage III or IV disease, suggesting that endometriotic tissues responds to androgens [54]. As shown in **Fig. 2**, FSHR activation recruits beta-arrestin2 for its desensitization and internalization [55]. Beta-arrestin2 inhibits AR directly and acts as a corepressor of AR by serving as a scaffold for Mdm2, leading to the ubiquitination and degradation of AR [56]. On the other hand, HSD17B3 may stimulate AR through the regulation of cytoplasmic testosterone metabolism [57].

Our results also support the role of AR in the pathophysiology and therapeutics of endometriosis. First, danazol, an androgen analog used for treatment of endometriosis, directly binds to the AR of endometriotic tissue [58] and decreases the expression of Bcl-2 (a suppressor for apoptosis) [59], resulting in the cell death of endometriotic tissue. Second, an animal study has shown that danazol *in vivo* reduces AR, estrogen receptors, and progesterone receptors of the endometrium [60]. Third, an *in vitro* study has demonstrated that the toxic effects on the endometrial stromal cells by danazol, such as destruction of cell organelles and cytoskeleton, were mainly mediated by androgen receptors [61].

Bioinformatics using databases in this investigation provided useful predictions for conformational changes of proteins affected by nsSNPs. Our results suggest that glycosylated 307^Thr^ of FSHR may mediate extracellular recognition events, which may be important in the development of endometriosis. In addition, the significant associations between the conversion of 680^Asn^ to 680^Ser^, resulted from A to G of rs6166, and endometriosis (**Tables 2** **to** **4**) suggest that phosphorylation of the cytoplasmic residue 680^Ser^ may be important for normal signaling pathways against the development of endometriosis. Furthermore, conversion of 289^Gly^ to 289^Ser^ (G to A of rs2066479) of HSD17B3 is associated with a decreased risk of endometriosis in younger women (**Table 3**). Although Ser/Gly of residue 289 of HSD17B3 was proposed to be a neutral polymorphism [62], we found that the phosphorylation of 289^Ser^ in HSD17B3 may decrease the risk of endometriosis.

Polymorphisms in promoter regions of genes have been shown to affect the levels of gene expression. For instance, promoter polymorphism of interleukin-10 gene (rs180087) was recently shown to be associated with the risk of endometriosis [63] **.** Polymorphisms in regulatory elements of genes may be localized at the site for methylation, which may change the susceptibility of gene silencing. On the other hand, non-synonymous SNPs in exons change the conformation of proteins, likely affecting protein functions, especially in an enzyme [27].

Inhibition of estrogen itself or estrogen-related steroid conversion pathways (**Fig. 1**) has been actively studied for the development of targeted therapy for endometriosis [5], [6]. It is conceivable that the designer’s drugs aiming at endometriosis-specific structural changes of key proteins may exert the greatest efficacy against disease but spare undesirable effects against enzymes with normal structures. Our results did not identify endometriosis-specific amino acid changes in HSD17B1 (**Table 2**) but detected endometriosis preferential structural changes of HSD17B3 (**Table 3**), CYP19 (**Table 3**), and FSHR (**Table 4** **,** **5** and **Fig. 3**). These findings may help us design disease-specific, targeted therapy. For instance, the extracellular domains of FSHR are theoretically targetable regions by drugs that are delivered by circulating blood. The higher prevalence of 307^Thr^ of FSHR (**Fig. 3**) makes it a highly rational target for drug development in endometriosis therapy. Similarly, drugs that aim at the domain of HSD17B3 containing 289^Gly^ may be more beneficial for the treatment of severe endometriosis that frequently occurs in young women (**Table 3**).

In conclusion, our results identified that 4 nsSNPs (rs6165, rs6166, rs2066479, rs700519) in estrogen synthesis and metabolism-related genes may decrease the risk of endometriosis. Because these 4 nsSNPs reside in 3 genes related to estrogen synthesis (HSD17B3, FSHR and CYP19) (**Fig. 1**), endogenous production of more estrogens, instead of slowing the degradation of estrogens and their metabolites, may be more strongly associated with the risk of endometriosis. Identification of the endometrosis-preferential nsSNPs and the conformational changes in those proteins may pave the way for the development of more disease-specific drugs in this devastating disease.

## Supporting Information

Table S1 **Serum levels of estradiol (E2) in patients with surgically confirmed endometriosis and age-matched healthy controls.**
(DOCX)

Table S2 **Genotypes and amino acid types/positions of non-synonymous single nucleotide polymorphisms (SNP) in estrogen synthesis and metabolism-related genes were obtained from National Center for Biotechnology Information (NCBI).** Status of polymorphism in the population studied and primers for the first polymerase chain reaction (PCR) and extension reaction at each SNP are also shown.
(DOC)

Database Links S1 **Supplementary Database Links.**
(DOC)
