# Supplementary material for: Functional Analyses of Endometriosis-Related Polymorphisms in the Estrogen Synthesis and Metabolism-Related Genes
Source: PLoS One. 2012 Nov 6;7(11):e47374. doi: 10.1371/journal.pone.0047374 (PMC3490981; doi:10.1371/journal.pone.0047374)
Supplement: Table S1 — Serum levels of estradiol (E2) in patients with surgically confirmed endometriosis and age-matched healthy controls. (DOCX) [file pone.0047374.s001.docx]

**Supplementary Table I.** Serum levels of estradiol (E2) in patients with surgically confirmed endometriosis and age-matched healthy controls.

|  |  | Endometriosis  (n=100) | | Age-matched control (n=100) | *P value* |
| --- | --- | --- | --- | --- | --- |
| Age | Mean ± SE* | 35 ± 1 | | 35 ± 1 | N.S. |
|  | Median | 35 | | 35 |  |
|  | Range | 17 - 52 | | 17 - 52 |  |
| Serum estradiol (pg/ml) |  | Pre-Op* | Post-Op* |  |  |
|  | Mean ± SE | 181 ± 19 | 178 ± 28 | 203 ± 35 | N.S. |
|  | Median | 117 | 103 | 141 |  |
|  | Range | 5 - 1121 | 5 - 2540 | 5 - 3386 |  |

* Abbreviation: SE, standard error; Pre-Op, before operation; Post-Op, after operation.
