# Supplementary material for: Functional Analyses of Endometriosis-Related Polymorphisms in the Estrogen Synthesis and Metabolism-Related Genes
Source: PLoS One. 2012 Nov 6;7(11):e47374. doi: 10.1371/journal.pone.0047374 (PMC3490981; doi:10.1371/journal.pone.0047374)
Supplement: Table S2 — Genotypes and amino acid types/positions of non-synonymous single nucleotide polymorphisms (SNP) in estrogen synthesis and metabolism-related genes were obtained from National Center for Biotechnology Information (NCBI). Status of polymorphism in the population studied and primers for the first polymerase chain reaction (PCR) and extension reaction at each SNP are also shown. (DOC) [file pone.0047374.s002.doc]

**Table S2.** Genotypes and amino acid types/positions of non-synonymous single nucleotide polymorphisms (SNP) in estrogen synthesis and metabolism-related genes were obtained from National Center for Biotechnology Information (NCBI). Status of polymorphism in the population studied and primers for the first polymerase chain reaction (PCR) and extension reaction at each SNP are also shown.

| Genes | SNP cluster ID. No. | Genotypes | Amino acid types | Amino acid types and positions | Status of polymorphism | Primers for the first PCR | | Primers for extension reaction |
| --- | --- | --- | --- | --- | --- | --- | --- | --- |
|  |  |  |  |  |  |  |  |  |
| *CYP19* | rs700519 | C/T | C:Arg T:Cys | Arg264Cys | polymorphism | sense | CAGCAAGGATTTGAAAGATGC | TTCTGATAGCAGAAAAAAGA |
|  |  |  |  |  |  | anti-sense | TGTGGCATGGGAATTACAGT |  |
| *CYP19* | rs2236722 | T/C | T:Trp C:Arg | Trp39Arg | polymorphism | sense | CCCTCTGAGGTCAAGGAACA | CTGGCCTTTTTCTCTTGGTG |
|  |  |  |  |  |  | anti-sense | CATGGACCAAAATCCCAAGT |  |
| *CYP19* | rs2304462 | A/G | A:His G:Arg | His264Arg | GG homozygous | sense | CAGCAAGGATTTGAAAGATGC | TCTGATAGCAGAAAAAAGAC |
|  |  |  |  |  |  | anti-sense | TGTGGCATGGGAATTACAGT |  |
| *CYP19* | rs1803154 | A/T | A:Lys T:Ter | Lys108Ter | AA homozygous | sense | CACATTGCATTTGGAGCAAC | CAAGTATGTTCCACATAATG |
|  |  |  |  |  |  | anti-sense | GATGCCTTTCTCATGCATACC |  |
|  |  |  |  |  |  |  |  |  |
| *CYP1A1* | rs1048943 | A/G | A:Ile G:Val | Ile462Val | polymorphism | sense | CTCACCCCTGATGGTGCTAT | AAAGACCTCCCAGCGGGCAA |
|  |  |  |  |  |  | anti-sense | TTTGGAAGTGCTCACAGCAG |  |
| *CYP1A1* | rs1799814 | A/C | A:Asn C:Thr | Asn461Thr | CC homozygous | sense | CTCACCCCTGATGGTGCTAT | GGAAGTGTATCGGTGAGA |
|  |  |  |  |  |  | anti-sense | TTTGGAAGTGCTCACAGCAG |  |
| *CYP1A1* | rs2229150 | C/T | C:Arg T:Trp | Arg93Trp | CC homozygous | sense | CTGGCACTGTCAAGGATGAG | AGCGGCCTGGACACCATC |
|  |  |  |  |  |  | anti-sense | AGAGTCTGGGCTGAAGGACA |  |
| *CYP1A1* | rs2278970 | C/G | C:Ala G:Gly | Ala463Gly | GG homozygous | sense | TTTGGAAGTGCTCACAGCAG | AGAAAGACCTCCCAGCGG |
|  |  |  |  |  |  | anti-sense | CTCACCCCTGATGGTGCTAT |  |
| *CYP1A1* | rs2856833 | A/C | A:Leu C:Phe | Leu381Phe | CC homozygous | sense | GACAGATCCCATCTGCCCTA | CTTCCGACACTCTTCCTT |
|  |  |  |  |  |  | anti-sense | AGGAAGCTCAGTCAGGCTCA |  |
| *CYP1A1* | rs4646422 | G/A | G:Gly A:Asp | Gly45Asp | polymorphism | sense | TCATGCTTTTCCCAATCTCC | ATCCACCAGGGCCATGGG |
|  |  |  |  |  |  | anti-sense | CCATACTGCTGGCTCATCCT |  |
| *CYP1A1* | rs4987133 | C/T | C:Thr T:Ile | Thr286Ile | GG homozygous | sense | GACCAGACCTGGATGGAGAG | CTACCTAAGGGCCACATC |
|  |  |  |  |  |  | anti-sense | AGGACACAATGGGGTAACCA |  |
|  |  |  |  |  |  |  |  |  |
| *CYP1B1* | rs10012 | C/G | C:Arg G:Gly | Arg48Gly | polymorphism | sense | AGACCACGCTCCTGCTACTC | CAACGGAGGCGGCAGCTC |
|  |  |  |  |  |  | anti-sense | CGCCATTCAGCACCACTAT |  |
| *CYP1B1* | rs1056827 | G/T | G:Ala T:Ser | Ala119Ser | polymorphism | sense | CCCCATAGTGGTGCTGAATG | GCCTTCGCCGACCGGCCG |
|  |  |  |  |  |  | anti-sense | CTGGCGCGTGAAGAAGTT |  |
| *CYP1B1* | rs1056836 | C/G | C:Leu G:Val | Leu432Val | polymorphism | sense | CCCAAGGACACTGTGGTTTT | TGAATCATGACCCA |
|  |  |  |  |  |  | anti-sense | GCCAGGATGGAGATGAAGAG |  |
| *CYP1B1* | rs1800440 | A/G | A:Asn G:Ser | Asn453Ser | AA homozygous | sense | CCCAAGGACACTGTGGTTTT | CTCTGCTGGTCAGGTCCTTG (anti-sense) |
|  |  |  |  |  |  | anti-sense | GCCAGGATGGAGATGAAGAG |  |
| *CYP1B1* | rs4398252 | C/T | C:Val T:Met | Val372Met | TT homozygous | sense | GGTAGCCCAAGACAGAGGTG | GGTTGGGCTGGTCACCCA |
|  |  |  |  |  |  | anti-sense | GCTCACTTGCTTTTCTCTCTCC |  |
| *CYP1B1* | rs4986887 | C/G | C:His G:Asp | His441Asp | GG homozygous | sense | CCCAAGGACACTGTGGTTTT | TAACCCGGAGAACTTT |
|  |  |  |  |  |  | anti-sense | GCCAGGATGGAGATGAAGAG |  |
| *CYP1B1* | rs4986888 | C/G | C:Ala G:Gly | Ala443Gly | CC homozygous | sense | CCCAAGGACACTGTGGTTTT | TCCTTGTCCAAGAATCGA (anti-sense) |
|  |  |  |  |  |  | anti-sense | GCCAGGATGGAGATGAAGAG |  |
|  |  |  |  |  |  |  |  |  |
| *HSD17B1* | rs605059 | G/A | G:Gly A:Ser | Gly313Ser | polymorphism | sense | AGACCCAGGGGACAAAGAAG | CTCAGGGTCCCCCACCGCAC |
|  |  |  |  |  |  | anti-sense | GAAGTGTTCGGCGACGTT |  |
|  |  |  |  |  |  |  |  |  |
| *HSD17B2* | rs8191136 | A/G | A:Thr G:Ala | Thr121Ala | GG homozygous | sense | CTCACTCCCTACTCCCCTGA | AATGAAAATGGCCCAGGA |
|  |  |  |  |  |  | anti-sense | TTATCTGCACTGGCTTCGTG |  |
| *HSD17B2* | rs8191246 | A/G | A:Ter G:Trp | Thr388Trp | polymorphism | sense | TATTACACGCCAGGGAAAGG | ACAAGAAAAAGGCCACCT |
|  |  |  |  |  |  | anti-sense | AACCCAGTTTCCCAGTTTCC |  |
|  |  |  |  |  |  |  |  |  |
| *HSD17B3* | rs2066479 | A/G | A:Ser G:Gly | Ser289Gly | polymorphism | sense | ATTCCTTCCTCCCCAACACT | GCCTGGGCCTTCTACAGC |
|  |  |  |  |  |  | anti-sense | GGTTGAAGTGCTGGTCTGCT |  |
| *HSD17B3* | rs2066480 | A/G | A:Ile G:Val | Ile31Val | GG homozygous | sense | GTCCCAGGAGGCATACACAG | GTGAGATTCTCCAGATGT |
|  |  |  |  |  |  | anti-sense | CCATTGACCGCAAGAAAGAC |  |
|  |  |  |  |  |  |  |  |  |
| *ERα* | rs9340773 | A/G | A:Ser G:Gly | Ser77Gly | GG homozygous | sense | CCGCCTACGAGTTCAACG | CAGGTCTACGGTCAGACC |
|  |  |  |  |  |  | anti-sense | TCCAGGTAGTAGGGCACCTG |  |
| *ERα* | rs17847065 | A/C | A:Gln C:Pro | Gln146Pro | CC homozygous | sense | CAGGTGCCCTACTACCTGGA | CGGTGCGCGAGGCCGGCC |
|  |  |  |  |  |  | anti-sense | CGGCGAGAGAACTTGACTCT |  |
| *ERα* | rs17847076 | A/T | A:Thr T:Ser | Thr168Ser | AA homozygous | sense | CCCAGGCCAAATTCAGATAA | GGCAGAGAAAGATTGGCCAGT |
|  |  |  |  |  |  | anti-sense | CAGTCGCTTTGGCTCTTAGG |  |
|  |  |  |  |  |  |  |  |  |
| *FSHR* | rs6165 | A/G | A:Thr G:Ala | Thr307Ala | polymorphism | sense | ACCCTGCACAAAGACAGTGA | GTTGATTATATGACTCAG |
|  |  |  |  |  |  | anti-sense | CGTCATGTCAAATCCTCTGC |  |
| *FSHR* | rs6166^#^ | A/G | A:Asn G:Ser | Asn680Ser | polymorphism | sense | GCAAGTGTGGCTGCTATGAA | CAGCTCCCAGAGTCACCA |
|  |  |  |  |  |  | anti-sense | CCCTTCAAAGGCAAGACTGA |  |
| *FSHR* | rs6167 | C/G | C:Ser G:Arg | Arg524Ser | CC homozygous | sense | CTTTGGCATCAGCAGCTACA | GCCCATGGATATTGACAG |
|  |  |  |  |  |  | anti-sense | GCCATGCAGAGGAAGTCAGT |  |
| *FSHR* | rs1126714 | A/C | A:Asn C:Thr | Asn112Thr | CC homozygous | sense | TGAGTGGATGGGATGAATGA | ACAACCTGCTCTACATCA |
|  |  |  |  |  |  | anti-sense | TGGGGGTACCAAACTACATGA |  |
|  |  |  |  |  |  |  |  |  |
| *COMT* | rs6270 | C/G | C:Ser G:Cys | Ser34Cys | GG homozygous | sense | CTGCAGGAGGAGCACAGAG | ACTGGGGCTGGGGCCTGT |
|  |  |  |  |  |  | anti-sense | GTGTCACCCATGAGCAGGTT |  |
| *COMT* | rs6267 | G/T | G:Ala T:Ser | Ala72Ser | GG homozygous | sense | ACAACCTGCTCATGGGTGAC | CATGCGGAGCCCGGGAAC |
|  |  |  |  |  |  | anti-sense | TCCTGTAAGGGCTTTGATGC |  |
| *COMT* | rs5031015 | A/G | A:Thr G:Ala | Thr102Ala | GG homozygous | sense | CACCTGTGCTCACCTCTCCT | AGGCAAGATCGTGGAC |
|  |  |  |  |  |  | anti-sense | CCCTTTTTCCAGGTCTGACA |  |
| *COMT* | rs4986871 | C/T | C:Ala T:Val | Ala146Val | CC homozygous | sense | CACCTGTGCTCACCTCTCCT | AGATCAACCCCGACTGTG |
|  |  |  |  |  |  | anti-sense | CCCTTTTTCCAGGTCTGACA |  |
| *COMT* | rs4680 | G/A | G:Val A:Met | Val158Met | polymorphism | sense | CACCTGTGCTCACCTCTCCT | GGATGGTGGATTTCGCTGGC |
|  |  |  |  |  |  | anti-sense | CCCTTTTTCCAGGTCTGACA |  |
|  |  |  |  |  |  |  |  |  |

*CYP19*, aromatase; *CYP1A1*, cytochrome P450IAI; *CYP1B1*, cytochrome P450IBI; *HSD17B1*, 17β-hydroxysteroid dehydrogenase I;

*HSD17B2*, 17β-hydroxysteroid dehydrogenase II; *HSD17B3*, 17β-hydroxysteroid dehydrogenase III; *ERα*, estrogen receptor α;

*FSHR*, FSH receptor; *COMT*, catechol-O-methyl transferase.

^#^ Data used were published previously in Wang *et al.*, 2011 .
