# Supplementary material for: Functional Analyses of Endometriosis-Related Polymorphisms in the Estrogen Synthesis and Metabolism-Related Genes
Source: PLoS One. 2012 Nov 6;7(11):e47374. doi: 10.1371/journal.pone.0047374 (PMC3490981; doi:10.1371/journal.pone.0047374)
Supplement: Database Links S1 — Supplementary Database Links. (DOC) [file pone.0047374.s003.doc]

**Supplementary Information for:**

**Functional Analyses of Endometriosis-Related Polymorphisms in the Estrogen Synthesis and Metabolism-Related Genes**

Hsin-Shih Wang^a,b^, Hsien-Ming Wu^a^, Bi-Hwa Cheng^b,c^, Chih-Feng Yen^a^, Pi-Yueh Chang^d^, Angel Chao^a^, Yun-Shien Lee^e,f^, Hsien-Da Huang^g*^, Tzu-Hao Wang^a,e*^.

^a^ Department of Obstetrics and Gynecology, Chang Gung Memorial Hospital, Lin-Kou Medical Center, Chang Gung University, Taoyuan, Taiwan;

^b^ Graduate Institute of Clinical Medical Sciences, College of Medicine, Chang Gung University, Taoyuan, Taiwan;

^c^ Department of Obstetrics and Gynecology, Chang Gung Memorial Hospital, Kaohsiung Medical Center, Kaohsiung, Taiwan;

^d^ Department of Laboratory Medicine, Chang Gung Memorial Hospital, Lin-Kou Medical Center, Taoyuan, Taiwan;

^e^ Genomic Medicine Research Core Laboratory, Chang Gung Memorial Hospital, Lin-Kou Medical Center, Taoyuan, Taiwan;

^f^ Department of Biotechnology, Ming Chuan University, Taoyuan, Taiwan;

^g^ Department of Biological Science and Technology, Institute of Bioinformatics and Systems Biology, National Chiao Tung University, HsinChu, Taiwan

**Content of supplementary information:**

**1. Supplementary Tables**

**2. Supplementary Database Links**

**Supplementary Database Links**

**FSHR_HUMAN**, Follicle-stimulating hormone receptor

<http://dbptm.mbc.nctu.edu.tw/search_result.php?search_type=db_id&swiss_id=FSHR_HUMAN>

<http://www.uniprot.org/uniprot/P23945>

***non-synonymous SNP***

1.     amino acid 680 of FSHR is Asn (instead of Ser),

<http://ca.expasy.org/cgi-bin/variant_pages/get-sprot-variant.pl?VAR_013905>

2.     the combination of a.a. 307 Thr (instead of Ala) and a.a. 680 Asn (instead of Ser) of FSHR.

<http://ca.expasy.org/cgi-bin/variant_pages/get-sprot-variant.pl?VAR_013903>

**HSD17B3**, Testosterone 17-beta-dehydrogenase 3

<http://dbptm.mbc.nctu.edu.tw/search_result.php?search_type=db_id&swiss_id=DHB3_HUMAN>

<http://www.uniprot.org/uniprot/P37058>

***non-synonymous SNP***

1.     amino acid 289 of HSD17B3 is Gly (instead of Ser),

<http://ca.expasy.org/cgi-bin/variant_pages/get-sprot-variant.pl?VAR_014871>
